# Optimizing physicochemical properties, antioxidant potential, and antibacterial activity of dry ginger extract using sonication treatment

**DOI:** 10.1016/j.heliyon.2024.e36473

**Published:** 2024-08-17

**Authors:** Nashi K. Alqahtani, Zakaria A. Salih, Saeed A. Asiri, Azhari Siddeeg, Sami A.D. Elssiddiq, Tareq M. Alnemr, Hosam M. Habib

**Affiliations:** a Research and Training Station, King Faisal University, P.O. Box 400, Al-Ahsa, 31982, Saudi Arabia; b Date Palm Research Center of Excellence, King Faisal University, P.O. Box 400, Al-Ahsa, 31982, Saudi Arabia; c Department of Food and Nutrition Sciences, College of Agricultural and Food Sciences, King Faisal University, P.O. Box 400, Al-Ahsa, 31982, Saudi Arabia; d Department of Food Engineering and Technology, Faculty of Engineering and Technology, University of Gezira, Wad-Medani, Sudan; e Research & Innovation Hub, Alamein International University (AIU), Alamein City, 5060310, Egypt

**Keywords:** Sonication, Physicochemical properties, Antioxidant assay, Antibacterial activity, Dry ginger extract

## Abstract

This research paper focused on enhancing the physico-chemical attributes, antioxidant capacity, and antibacterial effectiveness of dry ginger extract through sonication as an assistant extraction treatment. Ginger, resulting from the rhizome of *Zingiber officinale* Roscoe, is known for its culinary and medicinal uses outstanding to its antioxidant and antimicrobial possessions from phenolic acids and flavonoids. The study explored the use of sonication as an assistant extraction method and found that it significantly augmented the total phenolic content of the ginger extract by 28 % compared to traditional extraction methods, reaching 10.55 ± 1.50 mg GAE/g, DW. The research assessed the physicochemical belongings, antioxidant action, and antibacterial possibility of the sonicated ginger extract. The sonicated extract exhibited scavenging activity against the DPPH radical of 56.0 %. Pearson correlation investigation revealed a strong confident correlation between the phenolic content and antioxidant activity (r = 0.92, p < 0.01), as well as volatile compounds exhibited a moderate confident correlation with antibacterial action (r = 0.67, p < 0.05). The sonicated ginger extract also demonstrated potent antibacterial action, preventing the growth of both Gram-positive and Gram-negative bacteria. These findings contribute to the development of more efficient methods for extracting phenolic from ginger and provide insights into the relationships between phenolic and bioactive properties.

## 1 Introduction

Ginger, resulting from the rhizome of *Zingiber officinale* Roscoe from the Zingiberaceae family, is a highly popular spice with various culinary and medicinal uses [1,2]. It is renowned for its flavor-enhancing properties in food and beverages, as well as its therapeutic benefits [3]. The antimicrobial properties of ginger extracts make them effective in preventing foodborne epidemics, making them a valuable addition to food products [4,5]. Ginger is also recognized for its antioxidant activity, primarily attributed to its rich content of phenolic acids and flavonoids, which are secondary plant metabolites known for their antimicrobial and antioxidant possessions. By neutralizing harmful free radicals, antioxidants help to prevent cellular damage and slow down the aging process [6,7]. Additionally, antioxidants prevent lipid oxidation, thus averting food spoilage and rancidity [8,9]. Flavonoids, derived from phenylalanine, act as potent scavengers of free radicals and have garnered interest for their high antioxidant capacity, as verified in both *in vitro* and *in vivo* studies [[10], [11], [12]]. These compounds have been linked with a reduced risk of cardiovascular disease, anti-cancer through the activation of p53 to induce apoptosis [13], and age-related ailments [[14], [15], [16], [17]]. Therefore, ginger's antioxidant and antimicrobial possessions make it a capable natural alternative to chemical food preservatives [18]. Moreover, governments worldwide, principally in the European Union, are encouraging production industries to approve greener practices, prompting the need for more environmentally friendly food preservatives [19]. To harness the antioxidant and antimicrobial compounds existing in ginger efficiently, an effective extraction method is crucial. Old methods such as shaking extraction, Soxhlet extraction, and organic solvent extraction have limitations concerning solvent volume and extraction time, which are unfavorable from an environmental perspective [[20], [21], [22]]. Sonication, a novel and disruptive technology, has shown promise in extracting phenolic and other valuable compounds from agricultural products [23]. Ultrasonic waves induce cavitation, the development of bubbles within a liquid medium, in an aqueous environment [24,25]. The automatic vibrations in the ultrasonication expedient generate pressure waves that result in cavitation, with bubbles growing and coalescing as they rise to the surface due to Bjerke's forces. The bubbles can also increase and collapse through the compression of the wave, leading to highly localized temperatures (up to 5000 K) and densities of 1000 atm [22]. This disorderly nature of ultrasonication is utilized to enhance the extraction efficiency of phenolic from ginger. However, as ultrasonication is still a comparatively new technology and not yet universally scaled for all applications, careful design and development are necessary to enhance the extraction process. Given the widespread use of ginger and its significance as an herb, particularly in tea and coffee flavors, it is worthwhile to explore more efficient methods, such as ultrasonic-assisted solvent extraction, to extract phenolic. To our knowledge, previous studies have primarily examined the volatile composition and antibacterial possessions of essential oils extracted from fresh ginger samples [26,27]. Therefore, the novelty of this research lies in assessing the physicochemical possessions, antioxidant action, and antibacterial potential of the extract obtained from dried ginger using ultrasonication to increase the efficiency of extraction compared to traditional extraction methods. In addition to investigating the relationships between the phenolic contented, antioxidant action, and the volatile compounds with the antibacterial action of the sonicated ginger extract.

## 2 Materials and methods

### 2.1 Raw material and agents

Dry ginger (1 kg) was collected from a resident market in Al Hufuf, Kingdom of Saudi Arabia. The ginger was milled and kept at 4 °C pending use. All other chemicals and reagents (ethanol, acetic acid, chloroform, KI solution, sodium thiosulfate, aluminum chloride, gallic acid, sodium hydroxide, sodium nitrite, rutin, 2,2-Di(4-tert-octylphenyl)-1-picrylhydrazyl radical, Folin–Ciocalteu's phenol mixture, and dimethyl sulfoxide) were acquired from Sigma (St. Louis, MO, USA).

### 2.2 Extraction and ultrasonic treatment

The extraction process in the control treatment followed specific parameters. The solvent used was 90 % ethanol, and the extraction was directed at temperatures of 50 °C and 60 °C. The ginger powder quantity used was 40 g in 250 mL of solvent ethanol. The extraction process lasted for 20 min and 30 min for each temperature was used in the extraction method, with agitating every 2 min. After extraction, the solids were separated from the liquids by moving the flask contents into plugged tubes and centrifuging them at 3000 *g* for 10 min at 8 °C. The subsequent supernatants were calm and shifted to a round bottom flask. The solvent ethanol was then evaporated below a vacuum using a rotary evaporator at 50 °C. The dry extracts were kept at 4 °C until additional analysis.

In the Ultrasonic-assisted extraction treatment, an ultrasonic water bath (Bandelin, Sonorex, RK 510) with a temperature control unit was utilized. The water bath was a quadrilateral container measuring 300 mm × 240 mm × 150 mm, equipped with transducers on the lowest. It could generate a frequency of 35 kHz at a maximum power of 640W. For the extraction, the ginger powder was assorted with 250 mL of ethanol in a 1000 mL Erlenmeyer flask. The flask contents were then subjected to ultrasonication for different predetermined incubation times of 20 min and 30 min [28].

### 2.3 Physicochemical analysis

#### 2.3.1 Peroxide value

The peroxide value was measured following the technique described previously [29]. Firstly, 3 g of extract were balanced in a 250 mL conical flask. Then, 18 mL of a combination containing 3 parts acetic acid and 2 parts chloroform was added to the extracted tested sample. This was followed by the addition of 0.5 mL of saturated KI solution, and the test tubes were permitted to stand for 1 min. Afterward, approximately 100 mL of water and 3–4 drops of the starch indicator were added and thoroughly mixed. The samples were titrated against a standard 0.01 M sodium thiosulfate solution until the dark brown color disappeared. A blank test without the extract was also sonicated.

(1) $$\text{Oil}+\text{KI}+H_{2}O\to R-\text{OH}+H_{2}O+I_{2}+K^{+}$$

(2) $$I_{2}+2{\text{Na}}_{2}S_{2}O_{3}\;2\text{NaI}+{\text{Na}}_{2}S_{4}O_{6}$$

The peroxide rate of the sample was intended using the following equation:

(3) Peroxidevalue=((V1‐V0)×c×1000×T)/m

where:

V1 = The volume of sodium thiosulfate solution consumed in the main titration.

V0 = Amount of sodium thiosulfate solution consumed during the blank titration,

.

c = The molar concentration of the sodium thiosulfate solution.

T = The titer of the thiosulfate solution.

m = The mass of the weighed substance is denoted by m in grams.

#### 2.3.2 Determination of color

Color analysis was conducted using a CR-400 Chroma-Meter at room temperature (25 °C). The Hue angle (*h°*) was calculated using the formula:

(4) h°=tan/(b*/a*)

The color parameters, including a* (red-green axis), b* (yellow-blue axis), and luminosity (L*), were determined as previously described [30]. Total color difference (ΔE) was calculated based on L*, a*, and b* values using the equations:

(5) $$\Delta E=\sqrt{{\left(\Delta a\right)}^{2}+{\left(\Delta b\right)}^{2}+{\left(\Delta L\right)}^{2}}$$

(6) $$\Delta L^{*}=\Delta L_{\text{sample}}‐\Delta L_{\text{control}};\;\Delta a^{*}=a_{\text{sample}}‐a_{\text{control}}$$

(7) $$\Delta b^{*}=b_{\text{sample}}‐b_{\text{control}}$$

Chroma value (C∗) was calculated as reported previously:

(8) $$C*=\sqrt{a^{2}+b^{2}}$$

### 2.4 Volatile compounds analysis

Volatile compounds were analyzed using gas chromatography-mass spectrometry (GC-MS). A solid-phase microextraction (SPME) fiber was used to collect the compounds before GC-MS analysis [31]. Separation was achieved using a Varian model 3800 gas chromatograph equipped with a CP-Sil-8CB fused silica capillary column (30 m × 0.25 mm internal diameter, 0.25 μm film thickness). Alternatively, a DB WAX capillary column (30 m × 0.25 μm) from J&W Scientific was used.

The GC oven temperature was programmed as follows: initial hold at 40 °C for 3 min, ramp to 100 °C at 6 °C/min, and final ramp to 230 °C at 10 °C/min. The column flow rate was maintained at 0.9 mL/min. Mass spectra were acquired in electron impact (EI+) mode at 70 eV over a mass range of 33–450 *m*/*z*. Volatile compounds were identified by comparing their retention indices and mass spectra to those in the Wiley 130K and NIST 98 libraries (Agilent Technologies).

### 2.5 The total phenolic content (TPC)

The TPC of dry ginger extracts was determined using the Folin-Ciocalteu method. Initially, distilled water was added to a solution of ginger extract (100 μL) to achieve a final volume of 1 mL. Then, 0.5 mL of the Folin-Ciocalteu reagent, diluted 1:1 with water, and 2.5 mL of a 20 % w/v sodium carbonate solution were added to the mixture. The solution was thoroughly mixed and allowed to stand in the dark at room temperature for 40 min. Subsequently, the absorbance of the reaction mixture was measured at 725 nm using a spectrophotometer (Ultrospec 3100 pro, Amersham Biosciences, OR, USA) against a blank containing only the reagents.

The phenolic content of the ginger extract was expressed in milligrams of gallic acid equivalents (GAE) per gram of dry extract [32].

### 2.6 The Total Flavonoid Content (TFC)

The TFC of dry ginger extracts was determined using the aluminum chloride calorimetric method. Firstly, distilled water was added to the extract solution (100 μL) to achieve a final volume of 2 mL. Subsequently, a solution of sodium nitrite (0.15 mL, 5 % w/v) was added to the mixture. After incubating for 6 min, 0.15 mL of a 10 % w/v solution of aluminum chloride was added to the mixture and left to stand at room temperature for another 6 min. Then, a volume of 2 mL of a 4 % w/v sodium hydroxide solution, along with distilled water, was added to obtain a total volume of 5 mL. The reaction mixture was then allowed to stand in the dark for 15 min and its absorbance was measured at 510 nm using a spectrophotometer. To establish a calibration curve, rutin was used as the standard. The flavonoid contents in the ginger extract were expressed as milligrams of rutin equivalents (RE) per gram of dry extract [33].

### 2.7 HPLC quantification of phenolic acids and flavonoids

HPLC analysis was performed using a Waters HPLC system equipped with a 1525 Binary Pump, 2487 Dual UV Detector, and 717 Plus Autosampler. Separation was achieved using a Waters Xterra RP C18 column (5 μm, 4.6 × 150 mm) controlled by Breeze software (version 1.15). All HPLC-grade solvents were filtered through a 0.45 μm filter.

A linear gradient elution was employed using 1 % acetic acid (solvent A) and acetonitrile (solvent B). The gradient started at 5 % B, gradually increasing to 95 % B over 80 min, followed by a return to 5 % B and a 5-min post-run. The flow rate was 0.7 mL/min with a 20 μL injection volume. UV detection was monitored at 280 nm and 330 nm. All analyses were conducted at room temperature [33].

### 2.8 Antioxidant activity assay

The antioxidant capacity of the samples was assessed using the DPPH radical scavenging assay, adapted from Ref. [34]. Samples were incubated with a 300 μM DPPH solution in a 96-well plate at 37 °C for 30 min. DMSO was used to dissolve the samples, while DPPH was prepared in ethanol. The decrease in absorbance at 517 nm was measured using a spectrophotometer. The percentage inhibition of the DPPH radical was calculated as follows:

Percentage inhibition = [(Absorbance of control - Absorbance of sample)/Absorbance of control] x 100 (9)

### 2.9 Antibacterial assay

#### 2.9.1 Microorganisms and suspension preparation

The antibacterial activity was evaluated against four bacterial strains: *Bacillus subtilis* (NCTC 8236), *Staphylococcus aureus* (ATCC 25923), *Escherichia coli* (ATCC 25922), and *Pseudomonas aeruginosa* (ATCC 27853). Bacterial cultures were grown on nutrient agar slants for 24 h at 37 °C [35]. The resulting growth was harvested, washed with sterile saline, and suspended in saline to achieve a cell density of approximately 10⁸-10⁹ colony-forming units (CFU) per milliliter. This suspension was stored at 4 °C.

To determine the exact CFU count, serial dilutions of the suspension were prepared and plated on nutrient agar. After incubation at 37 °C for 24 h, colony counts were determined. The CFU per milliliter of the original suspension was calculated. Each new suspension was prepared under consistent conditions to maintain a similar CFU count.

#### 2.9.2 Antimicrobial susceptibility test

The antimicrobial activity of plant extracts was assessed using the disc diffusion method on Mueller Hinton agar (MHA) as described previously [33].

Bacterial suspensions were prepared to a concentration of 10⁸-10⁹ CFU/mL (equivalent to a McFarland standard of 0.5) and swabbed onto MHA plates. Sterile filter paper discs (6 mm diameter) impregnated with 20 μL of plant extract were placed on the inoculated plates. After 24 h of incubation at 37 °C, inhibition zone diameters were measured. Antimicrobial activity was categorized as follows: <9 mm (inactive), 9–12 mm (partially active), 13–18 mm (active), and ≥18 mm (very active) [36].

### 2.10 Statistical analysis

All experiments were conducted in triplicate, and data are presented as mean ± standard deviation. Statistical analysis was performed using SPSS version 26.0. One-way ANOVA followed by Tukey's post-hoc test was used to compare extraction rates among groups (p < 0.05). To assess the effect of sonication, an independent samples *t*-test was conducted (p < 0.05).

## 3 Results and discussion

### 3.1 Isolation of dry ginger extract using ultrasonic as assistant extraction method

Table 1, shows that the extraction rate was significantly influenced by both time and temperature, as well as the application of sonication. At the 20-min extraction time, the extraction rate was lower compared to the 30-min extraction time, regardless of the temperature. This indicates that a longer extraction time allowed for more efficient extraction of the ginger compounds. Regarding the effect of temperature, the extraction rate was higher at 60 °C compared to 50 °C, both for the un-sonicated and sonicated samples. This suggests that higher temperatures improve the extraction efficiency, likely due to enhanced solubility and mass transfer of the target compounds. The most notable observation is the effect of sonication. For all time and temperature conditions, the sonicated samples displayed a higher extraction rate compared to the un-sonicated samples. This demonstrates that the sonication treatment was effective in enhancing the extraction of compounds from the ginger powder. The combination of the optimal time (30 min) and temperature (60 °C) with the sonication treatment resulted in the highest extraction rate of 3.05 ± 0.93 %, which was significantly higher than the un-sonicated control (2.48 ± 0.11 %). These findings suggest that sonication-assisted extraction is a promising system for improving the yield and efficiency of ginger compound extraction, which could have important implications for the progress of ginger-based products with boosted functional possessions [37,38].

**Table 1:** Rate of dry ginger extract treated by ultrasonic.

Time (min)	Temperature (^o^C)	Extraction rate (%)	
		Un-sonicated	Sonicated
20	60	2.02 ± 0.21^b^*	2.35 ± 0.92^b^*
	50	1.66 ± 0.08^c^*	1.95 ± 0.88^c^*
30	60	2.48 ± 0.11^a^*	3.05 ± 0.93^a^*
	50	2.14 ± 0.19^b^*	2.77 ± 0.11^b^*

Data are presented as mean ± standard deviation. Different lowercase letters (a-c) within a column indicate significant differences (p < 0.05) between groups. An asterisk (*) within a row denotes significant differences (p < 0.05) between values.

The application of ultrasonic processing techniques in food extraction has been recognized for its minimal impact on food liquids. Ultrasonics is considered an innovative food extraction technology that has been widely acknowledged for its potential to improve food processing and improve quality [24]. The propagation of sound waves through liquid food media leads to a complex phenomenon known as “cavitation” [39]. The grouping of cavitation and heat exerts a significant result on the structure of heat-shocked cells, thereby increasing the effectiveness of enzyme and bacterial inactivation.

### 3.2 Physicochemical properties of dry ginger extract

Table 2 presents the analysis of peroxide value, and the analysis of color, comparing the sonicated dry ginger extract with the unsolicited sample.

**Table 2:** Color attributes of sonicated dry ginger extract compared with the un-sonicated sample.

Parameters	Sonicated sample	Un-sonicated sample
Peroxide value (meq O_2_·kg^−1^oil)	16.32 ± 0.9	16.89 ± 0.91
*L*∗	38.50 ± 0.91	37.90 ± 0.88
*a*∗	4.75 ± 0.89	4.60 ± 0.77
*b*∗	12.22 ± 0.33*	14.00 ± 0.21*
Hue	81.79 ± 0.31	81.86 ± 0.25
*C*∗	12.22 ± 0.50*	14.00 ± 0.29*
*ΔE*	0	0.09
Red	4.75 ± 0.95	4.60 ± 0.95
Yellow	12.22 ± 0.63*	14.00 ± 0.63*

^ΔE represents the color difference, while C^* ^indicates the chroma value. All measurements were performed in triplicate, with results expressed as mean±standard deviation. Significant differences between mean values within a row are indicated by an asterisk (^*^) (p<0.05) from each other.^

#### 3.2.1 Peroxide value

The ultrasonic treatment of dry ginger extract resulted in the lowest peroxide value of 16.3 meq O_2_·kg^−1^ oil, compared to the un-sonicated sample which had a peroxide value of 16.9 meq O_2_·kg^−1^ oil (Table 2). The peroxide value is an important indicator of rancidity in unsaturated fats and oils. It detects the presence of peroxide compounds that are formed during autoxidation, a free radical response linking oxygen that leads to the deterioration of fats and oils, resulting in off-flavors and off-odors. The concentration of peroxide in oil and fat is a valuable measure to assess the amount of degeneration [40].

The process of auto-oxidation involves the formation of peroxides as the main product, which contributes to offensive flavors in food products. This reaction occurs over a free radical chain reaction, where oxygen breaks the double bonds in fats and oils at room temperature. Nevertheless, photooxidation is a faster reaction that comprises the breaking of double bonds under light [41]. Rancidity in food substances can be attributed to both auto and photo-oxidation, which are natural oxidation and chemical degradation processes of oils. These processes convert fatty acid esters into free fatty acids, leading to the development of characteristic smells in many oils [38]. Oils with higher levels of unsaturation tend to undergo oxidation more rapidly than those with lower unsaturation levels [42]. The consequences of rapid oxidation in oils with higher levels of unsaturation include the development of unpleasant off-flavors and odors, a reduction in nutritional value, and a reduced shelf life of the oil. These changes can negatively impact the taste, aroma, and overall quality of the oil, making it less desirable for consumption or use in cooking and food preparation. Additionally, oxidized oils may pose health risks owing to the formation of potentially harmful compounds, such as free radicals and lipid peroxides. Therefore, it is important to handle and store oils with high unsaturation levels properly to minimize oxidation and preserve their freshness and nutritional benefits [43].

The results of the peroxide value analysis showed variations in all samples. This variability is due to the inherent instability of hydroperoxides formed during lipid oxidation, which break down into various volatile flavor compounds and nonvolatile products. Initially, the increase in peroxide value indicates a higher concentration of hydroperoxides, while a decrease suggests the occurrence of secondary oxidation products. The fluctuation in peroxide values is attributed to the rapid breakdown of primary oxidation products into secondary oxidation products [44].

#### 3.2.2 Color

Table 2 demonstrates that the values of red and yellow color in the dry ginger extract were higher for the sonicated samples, measuring 4.6 and 14.0 for red and yellow color, respectively, compared to 4.8 and 12.2 for the un-sonicated samples. This indicates the presence of pigments such as carotenoids and flavonoids in the ginger extract due to the ultrasonic treatment. Carotenoids and flavonoids are important compounds known for their contribution to the vibrant colors observed in various plant parts. Carotenoids, specifically, are natural pigments belonging to the class of isoprenoid compounds that impart yellow, orange, and red hues to plants. The composition of carotenoids can vary significantly among plant species and cultivars, with xanthophylls being the predominant type found in most plants' pale to deep yellow [45]. Thus, the common carotenoids in petals are xanthophylls, (the majority of carotenoids are xanthophylls**),** and the greatest of these xanthophylls are esterified with fatty acids. Significant differences (p < 0.05) were detected in the color values of the sonicated samples compared to the un-sonicated, except for the yellow color, *C∗*, and b* values. Therefore, under the given conditions, the ultrasonic behavior did not significantly affect t the color of the dry ginger extract. Previous studies have reported minimal color changes in sonicated food products, but it should be noted that the effect can become more pronounced with higher ultrasound parameters (intensity, duration, etc.) [46,47].

### 3.3 TPC and TFC of dry ginger extract treated by ultrasonic

According to Table 3, the sonicated dry ginger extract exhibited higher values of Total Phenolic Content (TPC) and Total Flavonoid Content (TFC) compared to the un-sonicated sample. The TPC was measured as 10.55 mg GAE/g DW extract for the sonicated sample, while it was 9.1 mg GAE/g DW extract for the un-sonicated sample. Similarly, the TFC was found to be 0.73 ± 0.01 mg RE/g DW extract for the sonicated sample, whereas it measured 0.72 ± 0.01 mg RE/g DW extract for the un-sonicated sample.

**Table 3:** Total phenolic content (TPC), total flavonoid content (TFC), and phenolic profile of dry ginger extract.

	Sonicated sample	Un-sonicated sample
TPC (mg GAE/g DW Extract)	10.55 ± 1.50*	9.10 ± 1.24*
TFC (mg RE/g DW Extract)	0.73 ± 0.01*	0.72 ± 0.01*
Gallic Acid (mg/100g)	130.49 ± 1.07*	125.77 ± 0.39*
Catechin (mg/100g)	216.04 ± 1.27*	209.22 ± 1.05*
Syringic Acid (mg/100g)	40.56 ± 1.40*	35.80 ± 0.34*
Caffeic Acid (mg/100g)	23.05 ± 0.70*	19.91 ± 0.66*
Rutin (mg/100g)	18.53 ± 0.89*	15.66 ± 0.40*
*p*-Coumaric Acid (mg/100g)	4.33 ± 0.43*	3.64 ± 0.21*
Ferulic Acid (mg/100g)	39.55 ± 0.64*	35.51 ± 0.46*
Quercetin (mg/100g)	58.08 ± 0.81*	54.31 ± 0.39*
Kaempferol (mg/100g)	14.11 ± 0.30*	11.44 ± 0.46*

Data are expressed as mean ± standard deviation. An asterisk (*) denotes statistically significant differences (p < 0.05) from each other between values within the same row.

The drying process plays a role in breaking down the cell wall of the food matrix and facilitating the release of phenolic compounds. In contrast, active enzymes present in fresh samples may degrade antioxidant compounds, depending on storage conditions, leading to a lower concentration of total phenolic and flavonoid content in fresh ginger [38]. However, in dried samples, the destructive enzymes are typically inactivated owing to the low water activity, allowing the retention of antioxidants in the dried extracts [37]. Additionally, Mustafa et al. [48], proposed that the drying process could lead to the development of new compounds with possible antioxidant capacity, increasing the overall antioxidant compounds.

### 3.4 Phenolic acids and flavonoids identified by HPLC

The analysis of the phenolic profile in dry ginger extract was conducted on both sonicated and un-sonicated samples identified by HPLC and presented in Table 3. Consistently, higher values were observed in the sonicated sample across various compounds. For example, the sonicated sample exhibited a greater content of gallic acid (130.49 ± 1.07 mg/100g) compared to the un-sonicated sample (125.77 ± 0.39 mg/100g). Gallic acid, known for its anti-inflammatory and antioxidant properties and found in a variety of plants and foods, has been studied for its potential in cardiovascular health and cancer prevention [49]. Similarly, Catechin showed values of 216.04 ± 1.27 mg/100g in the sonicated sample and 209.22 ± 1.05 mg/100g in the un-sonicated sample. Catechin is a type of flavonoid, which is abundant in teas and certain fruits. It is known for its antioxidant possessions, which may help defend cells from damage and reduce the risk of certain chronic diseases [50]. Moreover, syringic acid content was elevated in the sonicated sample (40.56 ± 1.40 mg/100g) compared to the un-sonicated sample (35.80 ± 0.34 mg/100g). Syringic acid, a natural phenolic acid with antioxidant and anti-inflammatory properties, has been researched for its possible benefits in cardiovascular health and as a protective agent against oxidative stress [51]. Moving on, caffeic acid levels were higher in the sonicated sample (23.05 ± 0.70 mg/100g) than in the un-sonicated sample (19.91 ± 0.66 mg/100g). Caffeic acid, present in various plant sources and known for its anti-inflammatory and antioxidant properties, has been investigated for its potential in reducing inflammation and protecting against certain diseases [52]. Furthermore, rutin content was increased in the sonicated sample (18.53 ± 0.89 mg/100g) compared to the un-sonicated sample (15.66 ± 0.40 mg/100g). Rutin, a flavonoid glycoside with antioxidant properties, is believed to have positive effects on blood circulation, cardiovascular health, and inflammation [53]. Additionally, *p*-Coumaric acid showed higher values in the sonicated sample (4.33 ± 0.43 mg/100g) than in the un-sonicated sample (3.64 ± 0.21 mg/100g). *p*-Coumaric acid, a hydroxycinnamic acid and potent antioxidant, may help defend cells from oxidative damage and decrease inflammation in the body [52]. Comparatively, ferulic acid content was greater in the sonicated sample (39.55 ± 0.64 mg/100g) than in the un-sonicated sample (35.51 ± 0.46 mg/100g). Widely distributed in plants, ferulic acid is a strong antioxidant with anti-inflammatory properties, studied for its potential to decrease the risk of chronic diseases like heart disease and cancer [52]. Similarly, quercetin levels were higher in the sonicated sample (58.08 ± 0.81 mg/100g) than in the un-sonicated sample (54.31 ± 0.39 mg/100g). Quercetin, a flavonoid with anti-inflammatory and antioxidant effects, is known for its potential to enhance heart health, lower blood pressure, and support immune function [54]. Lastly, kaempferol content was elevated in the sonicated sample (14.11 ± 0.30 mg/100g) compared to the un-sonicated sample (11.44 ± 0.46 mg/100g). Kaempferol, another flavonoid found in various fruits and vegetables, possesses anti-inflammatory and antioxidant properties and has been researched for its possibility to reduce the risk of chronic diseases [55].

The significant differences observed (p < 0.05) underscore the impact of sonication on phenolic extraction. Sonication, a widely utilized technique in various analytical methods for sample preparation, improves extraction efficiency and enhances the accessibility of target compounds [56,57]. It is important to note that these results are specific to the conditions and parameters used in this study. The efficacy of sonication treatment can differ based on factors such as sample composition, sonication time, and power intensity, which were determined through preliminary experiments to establish optimal temperature and time parameters. These conclusions are consistent with previous studies [[58], [59], [60]] regarding the concentration ranges of phenolic compounds.

### 3.5 Antioxidant activity assessment

The DPPH radical evaluation is widely used to evaluate the antioxidant activity of natural compounds by measuring the reduction of DPPH radicals in the presence of hydrogen-donating antioxidants. It is a sensitive method capable of detecting active components even at low concentrations and can process multiple samples efficiently within a short timeframe [61].

**Fig. 1:**
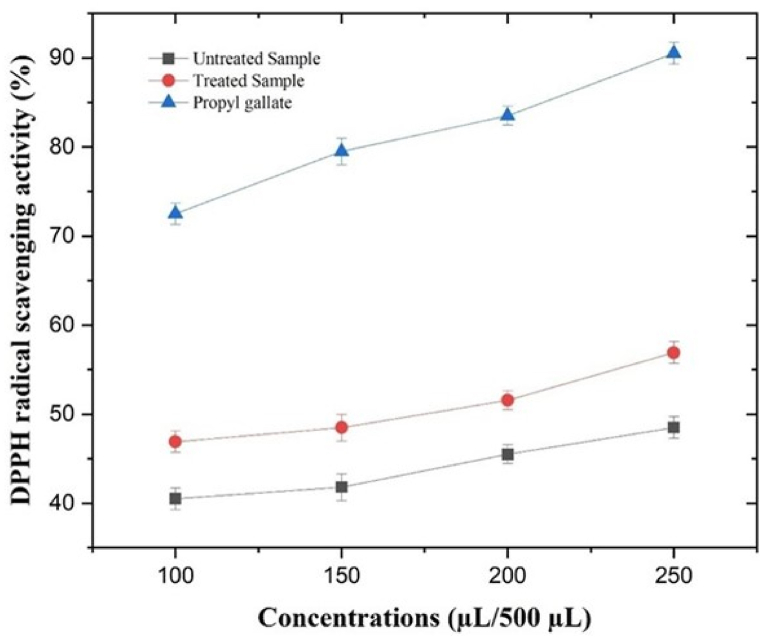
DPPH radical scavenging activity (%) of sonicated dry ginger extract and un-sonicated sample compared with a reference (Propyl gallate).

Fig. 1 illustrates the DPPH radical scavenging action of sonicated dry ginger extract compared to the un-sonicated sample, using Propyl gallate as a reference standard. The results indicate that the sonicated dry ginger extract exhibits stronger DPPH radical scavenging activity (56.0 %) at the highest concentration, in comparison to the un-sonicated sample (48.5 %). This difference in the result values of the standard (propyl gallate) compared with sonicated and un-sonicated samples could be attributed to the purity of the standard employed. It is worth noting that the scavenging activities of ginger extract tend to increase with higher concentrations of the phenolic.

The capability of antioxidants to scavenge DPPH radicals is attributed to their hydrogen-donating capacity [62]. The antioxidant activities of ginger extract derived from aromatic plants primarily stem from their active compounds. This can be attributed to the high percentage of major ingredients, as well as the presence of other ingredients in small quantities or the synergy among them [63].

**Fig. 2:**
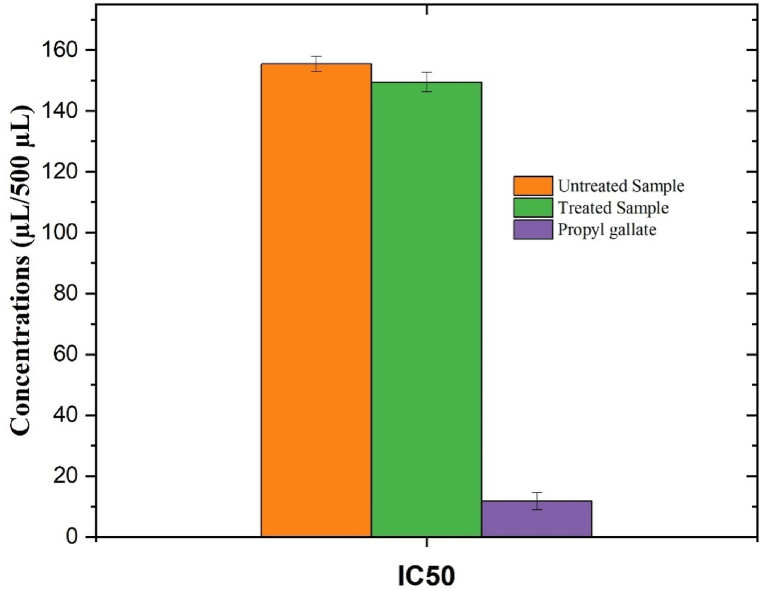
IC_50_ values of sonicated and un-sonicated dry ginger extract compared with the references.

Based on the reported results, the DPPH radical scavenging action and the standard curve of the dry ginger extract indicate an inhibition concentration of 50 % (IC_50_) of 149.5 μl/500 μl, which is lower than the IC_50_ value of the un-sonicated sample (155.5 μl/500 μl) (Fig. 1, Fig. 2). The DPPH radical scavenging action of thymol-rich essential oil from ginger which is considered an antioxidant compound. The radical scavenging activity of ginger's edible oil can be attributed to the presence of its main phenolic contents, particularly thymol, and their known effect on lipid oxidation in oils [64].

### 3.6 GC-MS volatile composition of dry ginger extract

Gas chromatography-mass spectrometry (GC-MS) analysis was conducted to examine the volatile composition of dry ginger after undergoing ultrasonic treatment. The results, presented in Table 4, indicate that the extract of sonicated dry ginger did not demonstrate a significant difference compared to the un-sonicated sample. However, both samples exhibited various functional groups of volatile compounds, including methyl, hydroxy, amine, flavone, ethyl, esters, and others. Notably, the sonicated extract revealed the presence of fifteen volatile constituents. The major constituents identified were 2,3-Butanediol, [R-(R*, R*)]- (12.59 % in untreated and 12.63 % in treated samples), which has been described to have anti-diabetic, antimicrobial, and neuroprotective effects. 4H-Pyran-4-one,2,3-dihydro-3,5-dihydroxy-6 (12.34 % in untreated and 12.35 % in treated samples) is known for its anti-inflammatory, antioxidant, and anti-cancer properties. The most abundant compound was [6]-Gingerol (18.81 % in untreated and 18.83 % in treated samples), a potent bioactive ginger constituent with well-documented anti-inflammatory, antioxidant, and anticancer activities.

**Table 4:** The volatile composition of the sonicated and un-sonicated dry ginger extract by using gas chromatography-mass spectrometry (GC-MS).

No	Constituent	Area (%)	
		Untreated sample	Treated sample
1	2,3-Butanediol, [R-(R*, R*)]-	12.59 ± 0.03	12.63 ± 0.01
2	2-Furanmethanol	1.38 ± 0.02	1.39 ± 0.02
3	Butyrolactone	5.91 ± 0.01	5.93 ± 0.02
4	2-Nonanol	1.77 ± 0.01	1.80 ± 0.01
5	1,3-Dioxolane, 2,4,5-trimethyl-	0.87 ± 0.03	0.89 ± 0.03
6	2,5-Dimethyl-4-hydroxy-3(2H)-furanone	8.94 ± 0.02	8.94 ± 0.03
7	4H-Pyran-4-one,2,3-dihydro-3,5-dihydroxy-6	12.34 ± 0.01	12.35 ± 0.02
8	2-Furanmethanol, 5-ethenyltetrahydro-. alpha	6.70 ± 0.03	6.74 ± 0.02
9	2-Butanone, 4-(4-hydroxy-3-methoxyphenyl)	7.22 ± 0.03	7.22 ± 0.03
10	Hexyl(2-heptyn-1-yl) amine	8.53 ± 0.02	8.14 ± 0.01
11	3,5-Octadiene, 2,2,4,5,7,7-hexamethyl-, (E, Z	3.27 ± 0.02	3.28 ± 0.01
12	(−)-Nortrachelogenin	5.52 ± 0.03	5.54 ± 0.02
13	[6]-Gingerol	18.81 ± 0.01	18.83 ± 0.03
14	Gingerol	3.74 ± 0.01	3.75 ± 0.03
15	Carinol	2.41 ± 0.01	2.56 ± 0.03

Other identified volatile compounds also possess notable health benefits. 2-Furanmethanol (1.38 % in untreated and 1.39 % in treated) has demonstrated antimicrobial and antioxidant activities. Butyrolactone (5.91 % in untreated and 5.93 % in treated) has been considered for its neuroprotective, anti-inflammatory, and anticancer properties. 2-Nonanol (1.77 % in untreated and 1.80 % in treated) has shown antimicrobial and antioxidant effects. 2,5-Dimethyl-4-hydroxy-3(2H)-furanone (8.94 % in both untreated and treated) exhibits antioxidant and anti-inflammatory activities. 2-Furanmethanol, 5-ethenyltetrahydro-. alpha (6.70 % in untreated and 6.74 % in treated) has been stated to have antimicrobial and antioxidant properties. Gingerol (3.74 % in untreated and 3.75 % in treated) is another important bioactive compound in ginger with anti-inflammatory, antioxidant, and anticancer effects [65].

Moreover, it is worth observing that the extract derived from ginger rhizomes has been found to prevent alterations in the levels of certain cytokines. This implies that these extracts exhibit immunomodulatory action and provide neuroprotection against inflammatory developments associated with neurodegeneration. The presence of volatile compounds in these extracts, which have been extensively studied for their anti-inflammatory properties and their ability to inhibit inflammatory mediators, may contribute to this activity [65].

The sonication treatment did not significantly distress the volatile composition of the ginger extract, with only minor changes observed in the relative percentages of some compounds, such as Hexyl(2-heptyn-1-yl) amine (8.53 % in untreated and 8.14 % in treated), indicating that the sonication process did not substantially alter the beneficial phytochemical profile of the ginger extract.

### 3.7 Antibacterial activity of the ginger extract

The antibacterial activity of the sonicated dry ginger extract was examined, and the results are presented in Table 5. It was observed that the extract derived from the sonicated dry ginger exhibited antibacterial properties against all tested bacterial strains. Specifically, the extract showed antibacterial possessions on *E. coli, P. aeruginosa, S. aureus,* and *B. subtilis*. The highest antibacterial activity was detected against *Bacillus subtilis*, with a mean zone of inhibition measuring 11 mm. On the other hand, *Staphylococcus aureus* exhibited the lowest mean zone of inhibition at 9.0 mm, followed by *Escherichia coli* and *Pseudomonas aeruginosa* with a mean zone of inhibition of 10.0 mm at a concentration of 100 mg/mL.

**Table 5:** Antibacterial activity of the sonicated and un-sonicated dry ginger extract.

Organism	(100 mg/mL)	
	Un-sonicated sample	Sonicated sample
*Escherichia coli*	10 ± 0.23*	11 ± 0.20*
*Pseudomonas aeruginosa*	10 ± 0.23	10 ± 0.90
*Staphylococcus aureus*	9 ± 0.23	9.75 ± 0.23
*Bacillus subtilis*	11 ± 0.23*	12.05 ± 0.23*

Data are presented as mean ± standard deviation. Significant differences between groups within the same row are indicated by an asterisk (*) (p < 0.05). Antimicrobial activity was categorized based on inhibition zone diameter: <9 mm (inactive), 9–12 mm (partially active), 13–18 mm (active), and >18 mm (very active).

These results are reliable with previous studies that have confirmed the antibacterial activities of ginger extract against both Gram-negative and Gram-positive bacteria [66]. The extent of inhibition activity was categorized based on the diameter of the inhibition zone, where a zone size below 9 mm indicated inactivity and a zone size between 9 and 12 mm indicated partial activity. The extract of dry ginger exhibited antibacterial activity against all tested bacteria, mostly Gram-positive strains [67,68].

Similar trends in the effect of ultrasonic treatment have been observed in previous studies on fresh ginger, albeit with different varieties [69,70]. Antibacterial agents are a group of ingredients that compete against pathogenic bacteria. Thus, by killing or reducing the metabolic activity of bacteria, their pathogenic consequence in the biological surroundings will be reduced and they are usually used for treating bacterial infections. Additionally, various antibacterial agents such as food additives and organic acids are utilized to inhibit foodborne bacteria and prolong the shelf life of treated foods [71]. Numerous naturally occurring compounds found in edible and medicinal plants, herbs, seeds, and spices have been exposed to have antibacterial properties and can serve as potential sources of antibacterial agents against food pathogens [72].

To the best of our knowledge, previous reports have focused on the volatile composition and antibacterial activity of essential oil extracted from fresh ginger samples. Thus, the novelty of this research lies in evaluating the antibacterial activity of the phenolic derived from dry ginger samples.

### 3.8 Correlation analyses

#### 3.8.1 Correlation between phenolic compounds and flavonoids among the antioxidant effects of sonicated dry ginger extract

**Fig. 3:**
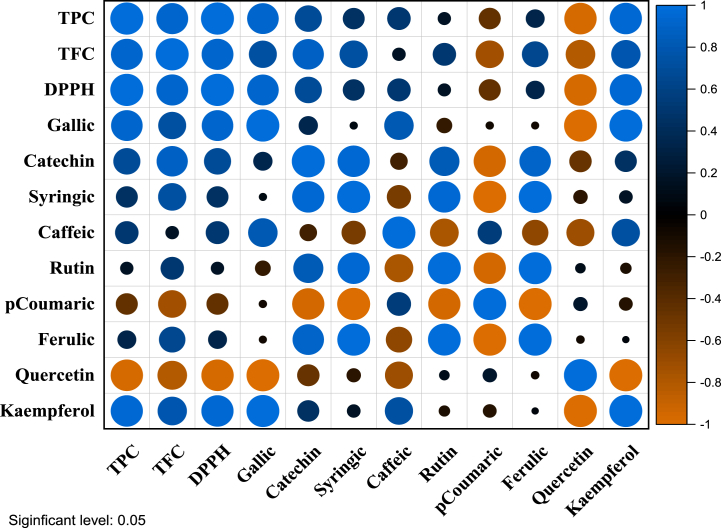
Relationship between phenolic content, composition, and antioxidant activity in dry ginger extract (visualized by correlation heatmap).

The purpose of this investigation was to investigate the impact of phenolic compounds and flavonoids on the antioxidant effects of sonicated dry ginger extract. To analyze the relationships among the variables, Pearson correlations were calculated and depicted in Fig. 3 as a correlation heatmap. The results revealed significant correlations among the different components.

Fig. 3 illustrates the Total Phenolic Content (TPC) of the extract exhibited strong positive correlations with Gallic Acid (*r*^*2*^ = 0.95), Catechin (*r*^*2*^ = 0.96), and Quercetin (*r*^*2*^ = 0.77). These findings suggest that higher TPC values are associated with increased levels of these compounds in the extract. This indicates that TPC can serve as a reliable indicator of the extract's antioxidant potential. Previous research has highlighted the importance of phenolic compounds in contributing to the antioxidant action of natural extracts, and the positive correlation between TPC and Gallic Acid is particularly notable. Gallic Acid is a well-known phenolic acid with potent antioxidant properties, and its strong positive correlation with TPC recommends that it may play a significant role in the extract's overall antioxidant potential. This conclusion is consistent with previous studies that have identified Gallic Acid as one of the key bioactive compounds in ginger.

Additionally, the Total Flavonoid Content (TFC) of the extract showed a strong positive correlation with DPPH radical (*r*^*2*^ = 0.99), indicating that higher TFC values are linked to greater DPPH radical scavenging activity. Flavonoids are known for their antioxidant properties, and the observed correlation suggests that the flavonoid content contributes significantly to the extract's antioxidant potential. This finding further emphasizes the importance of flavonoids in determining the antioxidant activity of the ginger extract.

Overall, the findings of this study demonstrate significant correlations between the phenolic and flavonoid content of the sonicated dry ginger extract and its antioxidant potential. These findings provide valuable insights for further research and support the notion that ginger extract rich in phenolic and flavonoid compounds may offer potential health benefits. Nevertheless, it is essential to recognize that additional studies are required to establish causal relationships and elucidate the underlying mechanisms responsible for these correlations.

#### 3.8.2 Correlation between volatile compounds and antibacterial activity of the sonicated dry ginger extract

**Fig. 4:**
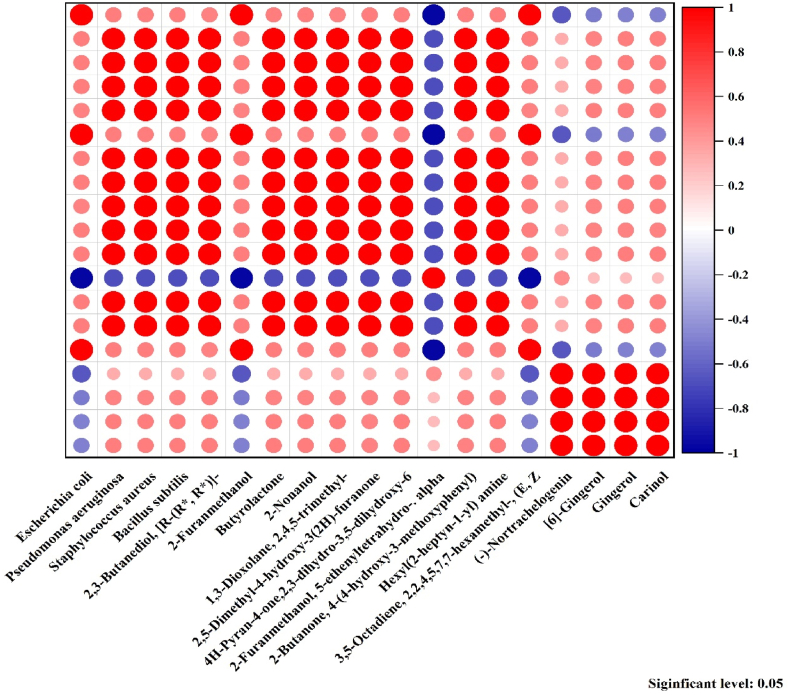
Relationship between volatile compound composition and antibacterial efficacy in sonicated dry ginger extract (visualized by correlation heatmap).

Fig. 4 shows Pearson correlation coefficients between various volatile compounds and bacterial species. *Escherichia coli* has a strong negative correlation (*r*^*2*^ = −0.971) with 2-Furanmethanol, and a strong positive correlation (*r*^*2*^ = 1.000) with 2-Furanmethanol.

5-ethenyltetrahydro-.alpha, and 1,3-Dioxolane, 2,4,5-trimethyl-. While, *Pseudomonas aeruginosa, Staphylococcus aureus, and* Bacillus *subtilis* all have very strong positive correlations (*r*^*2*^ = 1.000) with, 3. 2,3-Butanediol, [R-(R*,R*)]-, Butyrolactone, 2-Nonanol, 1,3-Dioxolane, 2,4,5-trimethyl-, 2,5-Dimethyl-4-hydroxy-3(2H)-furanone, and 4H-Pyran-4-one,2,3-dihydro-3,5-dihydroxy-6.

The strong positive correlations observed between certain bacterial species and volatile compounds suggest potential associations or interactions between them. For example, the high correlation between Escherichia coli and 2-Furanmethanol, 5-ethenyltetrahydro-.alpha indicates that the presence of this compound may be linked to the growth or activity of *E. coli.* Similarly, the widespread positive correlations between *Pseudomonas aeruginosa, Staphylococcus aureus, Bacillus subtilis,* and various other compounds could indicate that these bacteria have the ability to produce, utilize, or be affected by these volatile compounds. This information could be useful in understanding the metabolic processes and ecological relationships of these microorganisms. However, it's significant to note that correlation does not necessarily imply causation. Further investigation, such as experimental studies or additional data analysis, would be needed to determine the specific nature and directionality of the relationships between the bacterial species and volatile compounds.

## 4 Conclusion

This study demonstrated the efficacy of sonication-assisted extraction in optimizing the physicochemical possessions, antioxidant potential, and antibacterial activity of dry ginger extract. The sonicated ginger extract exhibited a higher yield, darker color, and enhanced bioactive properties compared to the non-sonicated control. The phenolic profile of the extract exposed the presence of key bioactive compounds, consisting of gingerol, shogaol, and paradol, which were positively correlated with the observed antioxidant and antibacterial activities. The conclusions of this study recommend that sonication is a promising technique for the efficient extraction of phytochemicals from ginger, resulting in a potent extract with improved functional and bioactive properties. The enhanced antioxidant and antibacterial actions of the sonicated ginger extract highlight its potential applications in the development of ginger-based functional foods, natural preservatives, and pharmaceutical formulations. Additional research is warranted to explore the scale-up potential of the sonication-assisted extraction process and to investigate the *in vivo* bioactivities and safety of the optimized ginger extract.

## Funding

This research was supported by a project (KFU241502) financed by the 10.13039/501100004686Deanship of Scientific Research, Vice Presidency for Graduate Studies and Scientific Research, King Faisal University, Saudi Arabia.

## Institutional review board statement

Not applicable.

## Informed consent statement

Not applicable.

## Data availability statement

All data analyzed or generated during this study are available within the manuscript.

## CRediT authorship contribution statement

**Nashi K. Alqahtani:** Visualization, Validation, Supervision, Resources, Project administration, Funding acquisition, Data curation, Conceptualization. **Zakaria A. Salih:** Writing – review & editing, Methodology, Formal analysis. **Saeed A. Asiri:** Writing – review & editing, Methodology, Formal analysis. **Azhari Siddeeg:** Writing – review & editing, Validation, Software, Methodology. **Sami A.D. Elssiddiq:** Writing – review & editing, Methodology, Investigation, Formal analysis. **Tareq M. Alnemr:** Visualization, Supervision, Software, Investigation, Data curation, Conceptualization. **Hosam M. Habib:** Writing – review & editing, Writing – original draft, Investigation.

## Declaration of competing interest

The authors declare that they have no known competing financial interests or personal relationships that could have appeared to influence the work reported in this paper.
