# Supplementary material for: Optimizing physicochemical properties, antioxidant potential, and antibacterial activity of dry ginger extract using sonication treatment
Source: Heliyon. 2024 Aug 17;10(16):e36473. doi: 10.1016/j.heliyon.2024.e36473 (PMC11382025; doi:10.1016/j.heliyon.2024.e36473)

**The chromatogram of the chemical composition of dry ginger extract treated by ultrasonic by using gas chromatography-mass spectrometry**


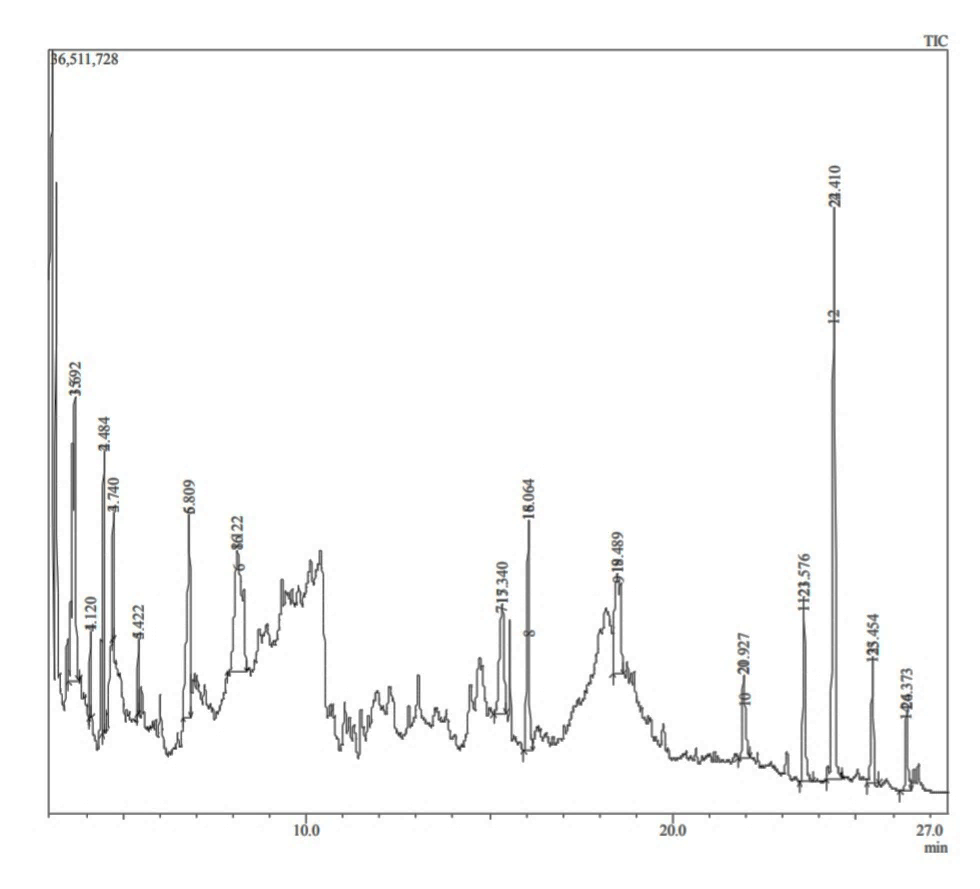

Supplement: Multimedia component 1 [file mmc1.docx]
